# An Efficient Strategy of Screening for Pathogens in Wild-Caught Ticks and Mosquitoes by Reusing Small RNA Deep Sequencing Data

**DOI:** 10.1371/journal.pone.0090831

**Published:** 2014-03-11

**Authors:** Lu Zhuang, Zhiyi Zhang, Xiaoping An, Hang Fan, Maijuan Ma, Benjamin D. Anderson, Jiafu Jiang, Wei Liu, Wuchun Cao, Yigang Tong

**Affiliations:** 1 State Key Laboratory of Pathogen and Biosecurity, Beijing Institute of Microbiology and Epidemiology, Fengtai District, Beijing, P. R. China; 2 College of Public Health and Health Professions, Emerging Pathogens Institute, University of Florida, Gainesville, Florida, United States of America; Columbia University, United States of America

## Abstract

This paper explored our hypothesis that sRNA (18∼30 bp) deep sequencing technique can be used as an efficient strategy to identify microorganisms other than viruses, such as prokaryotic and eukaryotic pathogens. In the study, the clean reads derived from the sRNA deep sequencing data of wild-caught ticks and mosquitoes were compared against the NCBI nucleotide collection (non-redundant nt database) using Blastn. The blast results were then analyzed with in-house Python scripts. An empirical formula was proposed to identify the putative pathogens. Results showed that not only viruses but also prokaryotic and eukaryotic species of interest can be screened out and were subsequently confirmed with experiments. Specially, a novel *Rickettsia* spp. was indicated to exist in *Haemaphysalis longicornis* ticks collected in Beijing. Our study demonstrated the reuse of sRNA deep sequencing data would have the potential to trace the origin of pathogens or discover novel agents of emerging/re-emerging infectious diseases.

## Introduction

Recently, researchers have used deep sequencing of small RNAto identify microRNAs that function in the transcriptional and post-transcriptional regulation of gene expression in plants and animals [1]and also to be an effective methods of virus discovery in plants and invertebrates [2]–[4]. This approach utilizes the mechanism in which small interfering RNAs are generated during the viral immunity process [5]. RNA silencing, or interference, as a form of viral immunity, begins with the recognition of a viral double-stranded or structured RNA by the Dicer nuclease family [6]–[8], which results in short interfering RNA (21–26 nt).

Due to the genomic diversity of differing pathogens, the current metagenomic approaches for microbial analysis require specific protocols to detect DNA viruses, RNA viruses, and other cellular pathogens [9], [10]. Because of this, sample processing is often labor intensive and costly. Since small RNA fractions could contain RNA metabolites derived from all RNA species, such as rRNAs, tRNAs, mRNA, snRNA, snoRNA [11], we hypothesize that it would be possible to use deep sequencing of sRNA as a universal strategy to identify multiple types of microorganisms other than viruses, including prokaryotic and eukaryotic pathogens. Therefore, in this study we demonstrate the use of sRNA deep sequencing method as a universal way to screen for multiple groups of pathogens, including viruses, bacteria, and eukaryotes, in wild-caught mosquitoes and ticks.

## Methods

### Collection of ticks and mosquitoes

**Figure 1:**
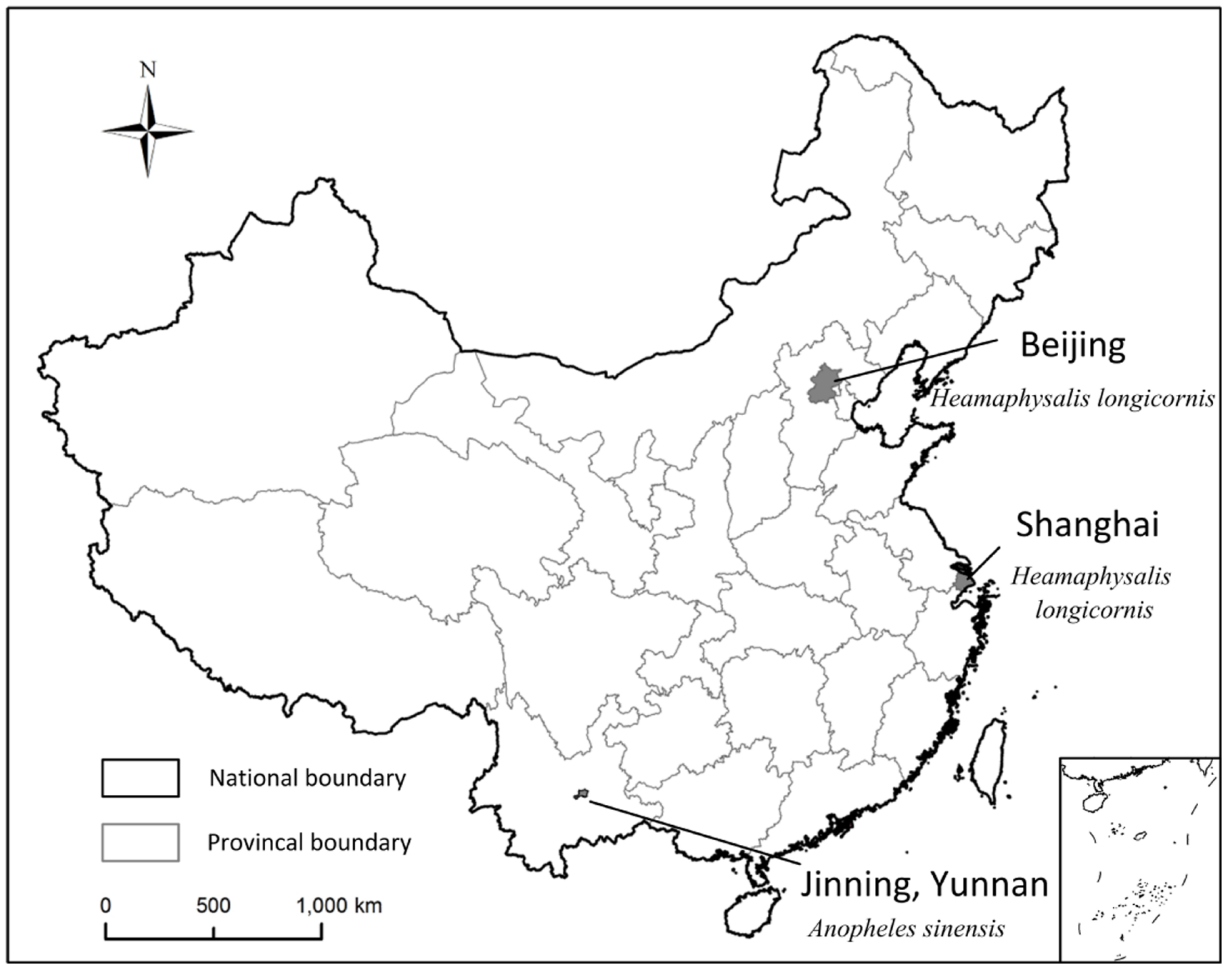
Areas from which ticks and mosquitoes in the study were collected.

Eight adult *Heamaphysalis longicornis* (*H. longicornis*) ticks were collected by dragging over the vegetation layer in the suburbs of Beijing, north of China (Fig. 1), pooled into one sample, and named XCP. Eighty-three larval *H. longicornis* ticks were hatched from eggs laid by adult ticks collected from Shanghai, east of China (Fig. 1), in 2011, pooled into one sample, and named CYP. Ticks were frozen at -80°C for 8 weeks until total RNA was extracted. Additionally, about 100 *Anopheles sinensis* (*A. sinensis*) mosquitoes were collected from Jinning, Yunnan province, southwest of China (Fig. 1), in 2009. Mosquitoes were stored in liquid nitrogen until total RNA was extracted. Morphologic features were observed under the anatomic microscope to identify the species and developmental stage of all tick and mosquito samples by entomologists (Y. Sun and R.M. Xu). The captured arthropods were not classified as endangered or protected species and were not privately owned. No specific permits were required for the described field studies.

### Small RNA library preparation, sequencing, and data cleaning

The two groups of ticks were disrupted in liquid nitrogen by pestles, from which total RNA was extracted using the Animal Tissue RNA Purification Kit (LC Sciences, Houston, TX, USA), according to the manufacturer's instructions. Mosquitoes were pooled, cleaned in sterilized water, and dried with hygroscopic filter paper. Total RNA was extracted using the Total RNA Purification Kit (LC Sciences, Houston, TX, USA), according to the manufacturer's instructions. The extracted total RNA was divided into two aliquots, one of which was sent for sRNA sequencing and the other was stored at −80°C for further testing.

Total RNA quality was then analyzed on an Agilent 2100 Bioanalyzer (Agilent Technologies, Palo Alto, CA, USA). Sequencing was conducted by LC Sciences (Houston, TX, USA), which included: 1) the brief purification of sRNA (18∼30 bp length) from the total RNA; 2) reverse-transcription into cDNA; 3) sequencing using the Illumina GAIIx machine. Following sequencing, the sRNA library was generated according to Illumina's sample preparation instructions.

The cleanup of the raw data was performed using a proprietary software package, AGGT101-miR v3.5 (LC Sciences, Houston, TX, USA). Briefly, the low-quality reads, simple artificial sequences, and adaptor sequences were removed. Unique sequences were then generated as clean data by collapsing the identical sequences, with the occurrence count of each unique sequence as a tag in the sequence name.

### Bioinformatics prediction

Each clean read was compared against the NCBI nucleotide collection (non-redundant nt database) using Blastn with default parameters, and the top 10 hits be outputted in the blast results. After filtering the hits with identity names containing “uncultured organism” or “ribosomal RNA”, and a match length less than 20 bp, the hit with the highest “Max Score” for every query was picked up. The resulting hits were grouped by genus according to its GI number.

In order to identify the most likely pathogens, the number of reads (read-number, RN) and the total matched length (match-length, ML) of each genus were calculated. To minimize the influence of non-specific matches, the total number of base pairs of every genus included in the nt database were counted (Nt-total, NT), and an empirical formula was derived based on the principle that the host species be ranked at the top of the genus list since its sRNAs should be dominant in the dataset. The empirical formula used was: Ratio = 1000×
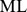
×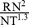. The results were ranked in descending order with the assumption that the higher a genus was ranked, the more likely it would exist in the sample. The putatively existing pathogenic species were supposed to be the species which are related with known pathogens and ranked highly (usually top-10) within every taxonomic domain. The sRNA sequences were then mapped with reference genomes of species of interest downloaded from GenBank using the program “Reference Assembly” of CLC Genomics Workbench (CLC bio, Aarhus, Denmark). All above-mentioned calculations were conducted with in-house Python scripts (available upon request).

### Confirmation with polymerase chain reaction (PCR)

Two sets of primers were used to amplify the genomic sequences of target species. The first sets of primers were designed using the reads from clean datasets mapped to reference genomes for detection of target species (Table 1). The second set of primers were adopted from previously reported amplification of 16s [12] or 18s rDNA [13] of targeted cellular species including *Rickettsia spp.*, *Coxiella spp.*, and *Aspergillus spp.* (Table 1). The total RNA stored at −80°C was reverse-transcribed using SuperScript III First-Strand Synthesis System (Invitrogen, Carlsbad, CA, USA) and the cDNA was used as a PCR template. Water was used as negative control. The target gene was amplified in 30 µL PCR mixtures containing 120 nM of each primer, 60 mM of each dNTP, 3 µL of 10× rTaq PCR buffer (Takara, Dalian, China), and 1.5 U of rTaq DNA polymerase (Takara, Dalian, China). PCR amplifications were conducted according to the manufacturer's instructions with annealing temperatures shown in Table 1. The PCR products amplified by specific primers were then directly sequenced using an ABI 3730 machine (Applied Biosystems, Foster City, CA, USA). For the sequencing results showing mixed sequences, the PCR products were cloned into the pGEM-T vector (Promega, Madison, WI, USA) and multiple clones were sequenced. The vector sequences were then trimmed off and the resulting sequences were compared against the NCBI nucleotide collection using Blastn with default parameters.

**Table 1:** Primers used in the study.

Sample (Target)	Primer Name	Primer	Annealing condition	Length
XCP (Rickettsia)	XCP-rick-2-1FXCP-rick-2-1R	GACGAAACAATCTCAGGAG TTAGAATAGAGGTTGCGG	50°C×4cycles, −1°C/step; 46°C×13cycles, −0.5°C/step; 40°C×35cycles	554 bp
	XCP-rick-2-2FXCP-rick-2-2R	GTGAGGGTGTTTCTGTTG CGAAATAGGATCGACGTG	46°C×13cycles, −0.5°C/step; 40°C×35cycles	138 bp
XCP (Coxiella)	XCP-cox-1-1FXCP-cox-1-1R	GGAGTGAATTGTACCAGA CCTACTCATTGTTACCCA	48°C×13cycles, −0.5cycles/step; 42°C ×35cycles;	959 bp
	XCP-Cox-1-2FXCP-Cox-1-2R	ATAAGGGTGAGGTCGGAA ATCATCGCTTGTTTGCCAG	48°C ×13cycles, −0.5cycles/step; 42°C ×35cycles;	421 bp
CYP (Coxiella)	CYP-Cox-2-1FCYP-Cox-2-1R	AGGAACAGTGTATGGTGG CTGAGTTCGGAATGGAAT	50°C ×4cycles, −1°C/step; 46°C ×13cycles, −0.5°C/step; 40°C×35cycles	777 bp
	CYP-Cox-2-2FCYP-Cox-2-2R	CTTGACTGCGAGACTGAC CAACGAATCTTTAGGGACC	46°C×13cycles, −0.5°C/step; 40°C×35cycles	476 bp
XCP&CYP (Coxiella)	Coxiella_spp_1_16s_FCoxiella_spp_1_16s_R	ATTGAAGAGTTTGATTCTGG CGGCTTCCCGAAGGTTAG	53°C×40cycles	1457 bp
XCP&CYP (universal 16s rDNA gene [12])	27F1492R	GAGAGTTTGATCCTGGCTCAG TACGGCTACCTTGTTACGAC	55°C×25cycles	1508 bp in *E.coli*
XCP&CYP (*Aspergillus* genus [13])	Asp1Asp2	CGGCCCTTAAATAGCCCGGTC ACCCCCCTGAGCCAGTCCG	53°C×38cycles	362 bp in *Aspergillus niger*
Mosquito (ESV)	ESVA-2-1FESVA-2-1R	CGAAGTGGACCAAGAAAGGAATGTA AATAAATCCCTGTCGAATCCAAAA	57°C×13cycles, −0.5°C/step;51°C×35cycles	572 bp
Mosquito (NDiV)	NDV-RdRp-1-3FNDV-RdRp-1-3R	ATGGTGCCCTCAGAAGTA GGTGTAGCGTTATTGAGTT	53°C×13cycles, −0.5°C/step;46°C×35cycles	529 bp

### Phylogenetic Analysis of target species

The gene used for phylogenetic analysis for *Coxiella spp.* and *Rickettsia spp.* was 16s rDNA, for *Aspergillus spp.* 18s rDNA, and for Nam Dinh virus (NDiV) the RNA-dependent RNA polymerase gene (RdRp gene) was used, all amplified using the PCR assay described above. The phylogenetic analysis (The GenBank Accession Numbers of the sequences were listed in Table S5) was performed using the Mega5 software (http://www.megasoftware.net). The alignment was done under default parameters. Phylogenetic analysis was performed by the Neighbor-joining method. All positions containing alignment gaps and missing data were deleted (complete-deletion). Amino acids translated from obtained nucleotide sequences were used for the phylogenetic tree construction for NDiV. The phylogenetic analysis of Espirito Santo virus(ESV) was reported in a previous comprehensive study [14].

## Results

### Pathogens Predicted in ticks and mosquitoes

For the sample CYP, the predicted top genus within the eukaryote domain was *Heamaphysalis spp.* (Table S1), with higher scores derived from the empirical formula than the predicted genus next to it. *Coxiella spp.* ranked second among the predicted bacteria (Table S2). Likewise, the top genus predicted from the sample XCP was *Heamaphysalis spp*. Of bacterial microbes, *Coxiella spp.* and *Rickettsia spp.* ranked as the top two and were the top predicted putative pathogens. No eukaryotic or viral pathogens were predicted in either XCP or CYP samples. The mapping percentage of *Coxiella spp.* and *Rickettsia spp.* are shown in Table 2. The wide-range random distribution of the reads, mapped onto the reference genome, suggests the existence of the predicted species, although the genome coverage percentage was low, which may be due to the large genome of the bacteria.

**Table 2:** Coverage of respective microbe genomes by mapped sRNA reads.

Sample	Species of pathogens	Read number	Base number	Genome coverage (%)
CYP	*Coxiella burnetii* (NC011528)	41316	969621	3.50
	*Aspergillus nidulans* FGSC A4 (BN001301)	64269	1159702	2.1
XCP	*Coxiella burnetii* (NC011528)	22702	533741	2.50
	*Rickettsia peacockii* (NC012730)	15576	369254	3.30
	*Aspergillus nidulans* FGSC A4 (BN001301)	47753	864327	1.5
*A. sinensis*	Espirito Santo virus (NC016518)	8891	202014	97.3
	Nam Dinh virus (NC015874)	7346	166439	83.5

For the sample of *A. sinensis*, no eukaryotic or prokaryotic pathogens were predicted. ESV, with a ratio of 2.94×10^9^, and NDiV, with a ratio of 1.29×10^8^, both showed a greater predictive likelihood as their ratios were much higher when compared to other viruses. The next closest predicted virus was Cavally virus, with a ratio of 2.52×10^6^ (Table S4). The considerably high read coverage of reference viral genome was obtained for ESV (97.3%, reference GenBank Accession: NC016518, NC016517) and NDiV (83.5%, reference GenBank Accession: NC015874). This finding also supported the increased likelihood of the sample containing these two viruses (Table 3).

**Table 3:** BLAST Results of confirmation experiment.

Sample	Primer	Target Genus/Species	Length	Top Hit Descriptions	Query Coverage	Max ID
Adult *H. longicornis* ticks from Beijing	XCP-Cox-1-2FXCP-Cox-1-2R	*Coxiella*	474 bp	*Coxiella burnetii* CbuG_Q212 (CP001019)	67%	98%
Adult *H. longicornis* ticks from Beijing	XCP-rick-2-2FXCP-rick-2-2R	*Rickettsia*	191 bp	*Rickettsia* montanensis str. OSU 85–930 (CP001019)	99%	98%
Nymphal *H. longicornis* ticks from Shanghai	CYP-Cox-2-2FCYP-Cox-2-2R	*Coxiella*	480 bp	*Coxiella burnetii* CbuG_Q212 (CP001019)	99%	98%
Adult *H. longicornis* ticks from Beijing	Asp1Asp2	*Aspergillus*	362 bp	Uncultured *Aspergillus* clone 1186 (DQ451600)	100%	100%
Nymphal *H. longicornis* ticks from Shanghai	Asp1Asp2	*Aspergillus*	362 bp	*Aspergillus candidus* strain CBS 567.65 (GU733348)	100%	100%
*A. sinensis*	NDV-RdRp-1-3FNDV-RdRp-1-3R	Nam Dinh virus	529 bp	Nam Dinh virus isolate SZ11714Z (CP001019)	100%	99%
*A. sinensis*	ESVA -2-1FESVA -2-1R	Espirito Santo virus segment A	572 bp	Espirito Santo virus segment A	98%	98%

### Validation of microbes by polymerase chain reaction

**Figure 2:**
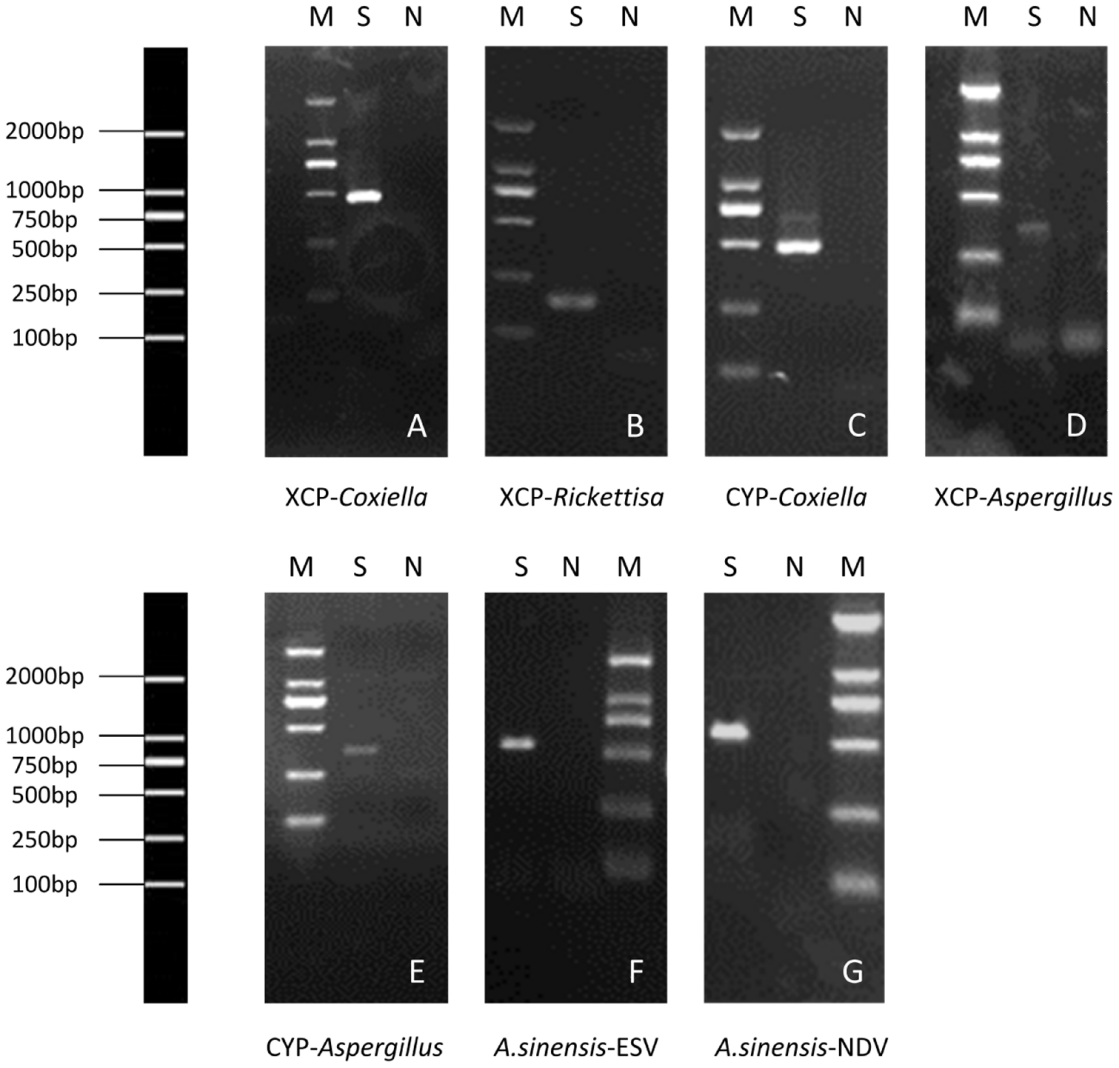
Experimental confirmation of predicted pathogens of interest predicted by bioinformatics. A), The second run of nested PCR amplification of the sample XCP (*Heamaphysalis longicornis* ticks collected from Beijing) to confirm the predicted *Coxiella* spp.. B), The second run of nested PCR amplification of the sample XCP (*Heamaphysalis longicornis* ticks collected from Beijing) to confirm the predicted *Rickettsia* spp.. C), The second run of nested PCR amplification of the sample CYP (*Heamaphysalis longicornis* ticks collected from Shanghai) to confirm the predicted *Coxiella* spp.. D), The PCR amplification of the sample XCP (*Heamaphysalis longicornis* ticks collected from Beijing) to confirm the predicted *Aspergillus* spp. E), The PCR amplification of the sample CYP (*Heamaphysalis longicornis* ticks collected from Shanghai) to confirm the predicted *Aspergillus* spp.. F), The PCR amplification of the sample *A. sinensis* collected from Yunnan to confirm the predicted ESV. G), The PCR amplification of the sample *A. sinensis* collected from Yunnan to confirm the predicted NDV. M, DNA marker; S, Sample; N,negtive control.

Target sequences were amplified in the validation experiments performed using PCR assays (Fig. 2). The determined sequences of these PCR products were subjected to BLAST analysis against the NCBI non-redundant nucleotide sequence database. Results confirmed the presence of *Coxiella spp.* in XCP and CYP, and *Rickettsia spp.* in XCP. Similarly, ESV and NDiV in *A. sinensis* were validated using the primers designed according to the sequences derived from sRNA sequencing. The blast results of the sequences are shown in Table 3.

As no eukaryotic pathogens were found likely to be in tick or mosquito samples, we instead chose to validate this strategy using *Aspergillus spp.* as it was the top predicted genus of fungi in the CYP sample and also ranked highly in the XCP sample (Table S3). Due to the small amount of reads mapped onto the relatively large *Aspergillus* genome, we were unable to design appropriate primer pairs according to the read sequences. Alternatively, we used a primer pair previously designed to diagnose invasive aspergillosis [13]. BLAST analysis demonstrated that the derived clones were homologous to *Aspergillus spp.* (Table 3) and therefore confirmed its presence in both samples.

### Phylogenetic Analysis of target species

**Figure 3:**
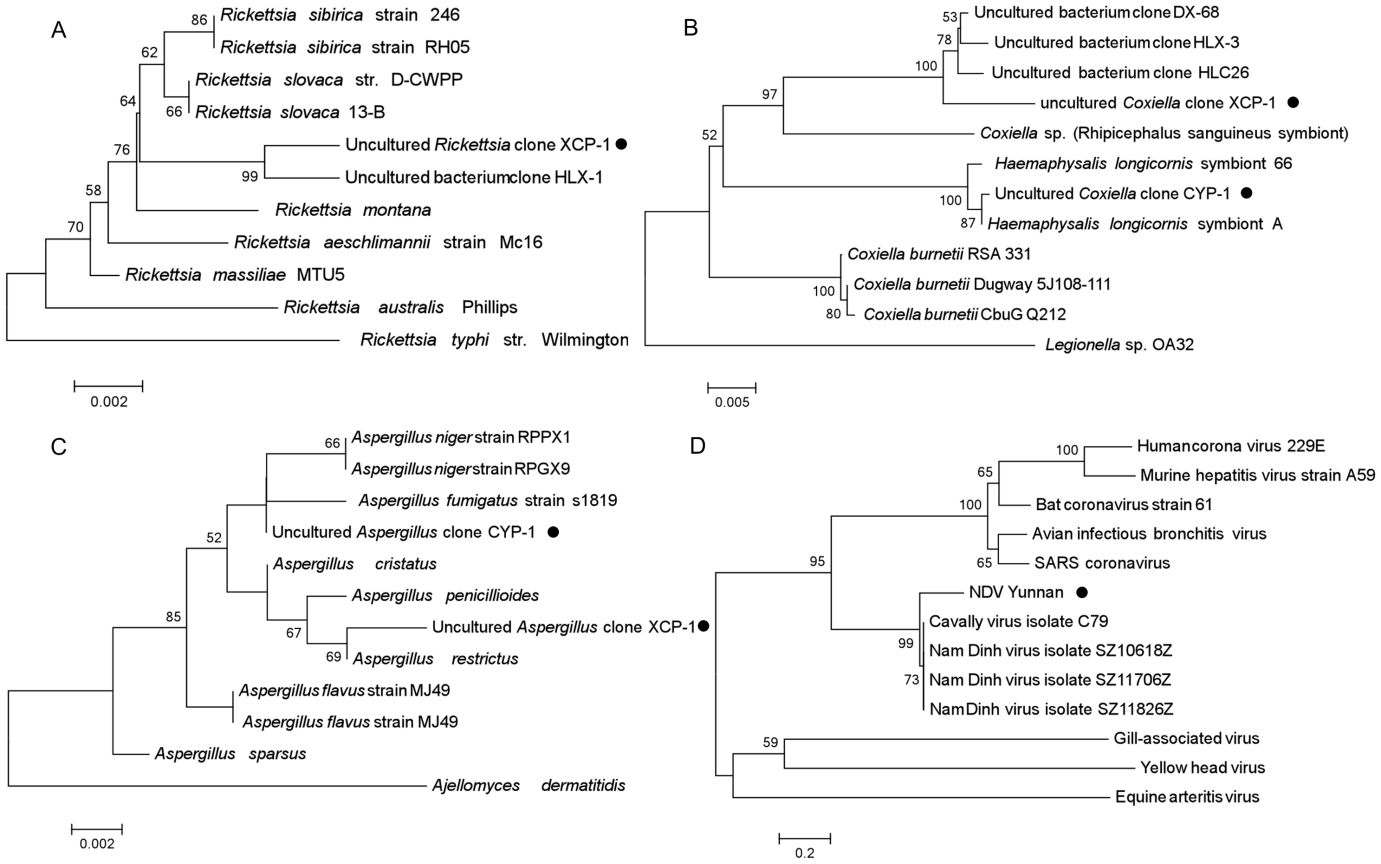
Phylogenetic analysis of confirmed pathogens of interests. Sequences were aligned using the MEGA5 (Version5.1) software package. NJ (Neighbor-joining method) phylogenetic tree construction and bootstrap analysis(1000 replicates) were carried out. Bars indicate the percentage of sequence divergence. All positions containing alignment gaps and missing data were deleted (Complete-deletion). **A.** Phylogenetic tree of bacteria belonging to *Rickettsia*, inferred from comparison of the partial 16s rDNA gene sequences. **B.** Phylogenetic tree of bacteria belonging to *Coxiella*, inferred from comparison of the partial 16s rDNA gene sequences. **C.** Phylogenetic tree of bacteria belonging to *Aspergillus*, inferred from comparison of the partial 18s rDNA gene sequences. **D.** Phylogenetic tree of bacteria belonging to *Nidovirales*, inferred from comparison of the partial RdRp gene sequences.

To explore the evolutionary status of identified pathogens of interest, we inferred dendrograms using the maximum parsimony method. The *Rickettsia spp.* identified in XCP was shown to be clustered within Rickettsia spotted fever group and most related with uncultured bacterium clone HLX-1 (JN866573) which was detected from *H. longicornis* collected in China (Fig. 3A). Our sequence and uncultured bacterium clone HLX-1 constituted a separate branch, indicating the presence of a uncharacteriased *Rickettsia* species in the XCP sample.

In the phylogenetic tree (Fig. 3B), the 16S rRNA gene amplified from the sample CYP was clustered with that of *H. longicornis* symbiont A, while differing from those of *Coxiella burnetii* strains (RSA 331, Dugway 5J108-111, CbuG Q212). The 16S rRNA gene of the XCP sample, was clustered with that of uncultured bacterium clone DX-68 (JN866592) from *Dermacentor silvarum* in China, uncultured bacterium clone HLX-3 (JN866574), and uncultured bacterium clone HLC26 (JN866567) from *H. longicornis* in China. *H. longicornis* symbiont A belongs to the genus *Coxiella* (Taxonomy ID: 776). The uncultured bacterium clones DX-68, HLX-3, HLC26 were in a cluster with that of *Coxiella spp.* (Rhipicephalus sanguineus symbiont, D84559), which was clustered with *C. burnetii* (CP000890, CP000733, CP001019). The result showed that the predicted *Coxiella spp.* in the sample CYP and XCP were in different clusters representing different species.

The phylogenetic analysis of *Aspergillus spp.* (Fig. 3C) demonstrated that our sequences were clustered with the *Aspergillus* species. The sequence amplified from sample XCP was clustered with *Aspergillus restrictus* , while the sequence amplified from CYP was closely related with that of *Aspergillus fumigatus* , *Aspergillus niger*.

For the phylogenetic analysis of NDiV (Fig. 3D), the RdRp gene was amplified and sequenced, as it is conserved in nidoviruses and suitable for use in evolutionary study [15]. Since the RdRp gene nucleotide sequences showed remarkably high diversity, making it difficult to find homology between them, we adopted the amino acid sequences of the RdRp genes to construct the phylogenetic tree. The result showed that the identified NDiV (KC776320, Nam Dinh virus isolate Yunnan) was clustered with different isolates of NDiV (JQ996713, JQ996715, JQ996712) and Cavally virus isolate C79 (HM746600), and was in different branches from *Coronaviridae* and *Arteriviridae*.

## Discussion

In the present study, we demonstrated that not only viruses, but also prokaryotic and eukaryotic pathogens could be screened out of samples using sRNA deep sequencing data.

For the discovery of viruses, our strategy described in this study showed a higher sensitivity of discovering viruses compared with our previous study. Huang *et al* [16] only reported the discovery of Mosquito X Virus (MXV) which is 97% identical to ESV in the same sRNA dataset *A. sinensis* as we use in this study. In that study, he used the sRNA data to BLAST only with the virus database, and no statistical analysis was applied.. In our study, the non-redundant nt database (NCBI) were used for BLAST procedure. Statistical analysis of blast results revealed the existence of another virus, NDiV in the sample and subsequent experiments confirmed the prediction.

More over, our strategy also showed the feasibility of screening pathogens of prokaryotes and eukaryotes. Specially, the *Rickettsia spp.* identified in *H. longicornis* captured in Beijing was shown to be most similar with uncultured bacterium clone HLX-1. Phylogenetic analysis of the 16S rRNA gene indicated it could likely be a novel species of *Rickettsia* spotted fever group (SFG) which unites a phylogenetically well-defined clade of Rickettsiae that are distinct from other species and that have a life cycle involving arthropods, mainly ticks [17]. SFG includes a number of pathogenic organisms that cause so-called tick-borne (TB) rickettsioses, which can cause diseases such as Rocky Mountain spotted fever in humans [18]. Additionally, many types of SFG rickettsia have been reported in Asia, Africa, North America, South America, Europe, and Australia [19], reinforcing the plausibility of this study's predicted *Rickettsia spp.* strain. In China, there are five species of tick-transmitted SFG rickettsiae that have all been isolated, named *R. sibirica*, [20], *R. mongolotimona* [21], *R. heilongjiangiensis*, *R. hulinii* [22], and BJ-90 strain [23]. Furthermore, molecular evidence of *R. raoultii* and *R. slovaca* has been reported in the northeast and northwest of China [24], [25]. Serological evidence of *Rickettsia japonica* [26], *Rickettsia conorii*, *Rickettsia akari* [27] has also been reported. The prediction of the genetic sequence of *Rickettsia spp.* in this study appears to be unique, suggesting a possible novel strain of the bacterium. This finding highlights that potential value this technique could have for species discovery.

*Coxiella spp.* and *Aspergillus spp.*, *from* samples XCP and CYP, were confirmed with additional experimentation. Interestingly, the discovery of *Coxiella spp.* in larval *H. longicornis* ticks, hatched from the eggs of adult ticks collected from Shanghai, indicated that transovarian and transtadial transmission of the *Coxiella* species had occurred consistent with that of a novel Coxiella-like agent [28].

However, non-specific match would occur in the prediction results. For example, from the *H. longicornis* ticks sample, *Heamaphysalis* was demonstrated as the top eukaryote among tested samples (Table S1). Amblyomma, Ixodes, Dermacentor, Aponomma were also in the top 10 genera of Eukaryota. Some irrelevant genera such as *Spirogyra* and *Psathyropus* also appeared in the top 10 genus of Eukaryota, but with short total matched lengths and small numbers of matched reads. These non-specific matches could have been caused by the following reasons: 1) the sequencing platform Illumina GAIIx had an inherent error rate roughly 0.1%–0.5%; 2) sequence homology exists between evolutionarily related species; 3) the BLAST program allows mismatches, which could result in false species assignments; 4) the sequence richness, diversity, and evenness of different species deposited in the nt database were biased. Due to the short length of the reads, these reasons described above would affect the predicted results.

Our study showed for the first time the capacity to screen for a variety of microbial pathogens, not just RNA [2] and DNA [29] viruses, but also prokaryotic, and eukaryotic pathogens, using the sRNA deep sequencing data obtained from wild-caught ticks and mosquitoes. Since microRNAs were originally discovered as critical regulators of developmental timing events in *Caenorhabditis elegans* [30], interest in understanding microRNAs has increased. Under this context, the sRNA deep sequencing technique was further developed and large amounts of sRNA deep sequencing data were derived from a variety of species. This readily available sRNA deep sequencing data can be used or re-used to find putative pathogens, which could be associated with a variety of known and unknown diseases. Our study showed the possibility of discovering pathogens by advanced mining of the sRNA deep sequencing data. This strategy of reuse of sRNA deep sequencing data with the ability of discovering all spectrums of microbial pathogens could have important application in pathogen screening, early warning and tracing the origins of emerging/re-emerging infectious diseases.

## Supporting Information

Table S1 **Top 10 genus of Eukaryota predicted from deep sequencing data of small RNAs.**
(DOCX)

Table S2 **Top 10 genus of Bacteria predicted from deep sequencing data of small RNAs.**
(DOCX)

Table S3 **Top 10 genus of Fungi predicted from deep sequencing data of small RNAs.**
(DOCX)

Table S4 **Top 10 genus of predicted viruses.**
(DOCX)

Table S5 **GenBank accession numbers of strains used in phylogenetic analysis.**
(DOCX)
