# Supplementary material for: An Efficient Strategy of Screening for Pathogens in Wild-Caught Ticks and Mosquitoes by Reusing Small RNA Deep Sequencing Data
Source: PLoS One. 2014 Mar 11;9(3):e90831. doi: 10.1371/journal.pone.0090831 (PMC3949703; doi:10.1371/journal.pone.0090831)
Supplement: Table S1 — Top 10 genus of Eukaryota predicted from deep sequencing data of small RNAs. (DOCX) [file pone.0090831.s001.docx]

**Table S1** Top 10 genus of Eukaryota predicted from deep sequencing data of small RNAs

| **Genus** | **Kingdom** | **Super Kindom** | **Nt-total** | **Match-length** | **Reads number** | **Ratio** | **Sample** |
| --- | --- | --- | --- | --- | --- | --- | --- |
| *Haemaphysalis* | Metazoa | Eukaryota | 339170 | 34923 | 16623 | 623717160 | CYP |
| *Amblyomma* | Metazoa | Eukaryota | 909667 | 15216 | 9263 | 23402198 | CYP |
| *Psathyropus* | Metazoa | Eukaryota | 2593 | 124 | 887 | 3558981 | CYP |
| *Ixodes* | Metazoa | Eukaryota | 23941092 | 121949 | 8675 | 2343312 | CYP |
| *Dermacentor* | Metazoa | Eukaryota | 405273 | 2932 | 3491 | 1832279 | CYP |
| *Aponomma* | Metazoa | Eukaryota | 30483 | 948 | 955 | 1280973 | CYP |
| *Bothriocroton* | Metazoa | Eukaryota | 63872 | 1641 | 517 | 248419 | CYP |
| *Rhipicephalus* | Metazoa | Eukaryota | 1580752 | 8043 | 1579 | 175251 | CYP |
| *Colpoda* | # | Eukaryota | 46390 | 841 | 472 | 160816 | CYP |
| *Mus* | Metazoa | Eukaryota | 5162665855 | 513503 | 32957 | 131735 | CYP |
| *Heamaphysalis* | Metazoa | Eukaryota | 339170 | 28098 | 9405 | 160638762 | XCP |
| *Amblyomma* | Metazoa | Eukaryota | 909667 | 12782 | 4572 | 4789212 | XCP |
| *Dermacentor* | Metazoa | Eukaryota | 405273 | 2939 | 5226 | 4115915 | XCP |
| *Aponomma* | Metazoa | Eukaryota | 30483 | 748 | 834 | 770830 | XCP |
| *Psathyropus* | Metazoa | Eukaryota | 2593 | 93 | 469 | 746252 | XCP |
| *Ixodes* | Metazoa | Eukaryota | 23941092 | 75808 | 5193 | 521993 | XCP |
| *Bothriocroton* | Metazoa | Eukaryota | 63872 | 1301 | 485 | 173323 | XCP |
| *Spirogyra* | Viridiplantae | Eukaryota | 409454 | 914 | 1292 | 77198 | XCP |
| *Rhipicephalus* | Metazoa | Eukaryota | 1580752 | 5543 | 984 | 46904 | XCP |
| *Oxyopes* | Metazoa | Eukaryota | 9300 | 65 | 254 | 29077 | XCP |
| *Culex* | Metazoa | Eukaryota | 29546421 | 4920782 | 872517 | 727655094132 | *A. sinensis* |
| *Demodex* | Metazoa | Eukaryota | 56101 | 660 | 16697 | 123355650 | *A. sinensis* |
| *Entomophaga* | Fungi | Eukaryota | 30523 | 1500 | 1942 | 8367088 | *A. sinensis* |
| *Aedes* | Metazoa | Eukaryota | 36557020 | 104797 | 12057 | 2243703 | *A. sinensis* |
| *Anopheles* | Metazoa | Eukaryota | 91635374 | 86988 | 14996 | 872425 | *A. sinensis* |
| *Gallus* | Metazoa | Eukaryota | 259431433 | 231786 | 13936 | 518960 | *A. sinensis* |
| *Mus* | Metazoa | Eukaryota | 5162665855 | 615708 | 39197 | 22343 | *A. sinensis* |
| *Danio* | Metazoa | Eukaryota | 1695530808 | 321107 | 24020 | 186079 | *A. sinensis* |
| *Entomophthora* | Fungi | Eukaryota | 65751 | 1126 | 550 | 185776 | *A. sinensis* |
| *Drosophila* | Metazoa | Eukaryota | 825422989 | 191951 | 17251 | 146265 | *A. sinensis* |

# No Rank
