# Supplementary material for: An Efficient Strategy of Screening for Pathogens in Wild-Caught Ticks and Mosquitoes by Reusing Small RNA Deep Sequencing Data
Source: PLoS One. 2014 Mar 11;9(3):e90831. doi: 10.1371/journal.pone.0090831 (PMC3949703; doi:10.1371/journal.pone.0090831)
Supplement: Table S2 — Top 10 genus of Bacteria predicted from deep sequencing data of small RNAs. (DOCX) [file pone.0090831.s002.docx]

**Table S2** Top 10 genus of Bacteria predicted from deep sequencing data of small RNAs

| **Genus** | **Kingdom** | **Super Kindom** | **Nt-total** | **Match-length** | **Reads number** | **Ratio** | **Sample** |
| --- | --- | --- | --- | --- | --- | --- | --- |
| *Acinetobacter* | # | Bacteria | 63631253 | 31091 | 7653 | 130475 | CYP |
| *Coxiella* | # | Bacteria | 11302775 | 7170 | 3810 | 70506 | CYP |
| *Comamonas* | # | Bacteria | 7172136 | 11389 | 1554 | 33655 | CYP |
| *Pseudomonas* | # | Bacteria | 270076122 | 51891 | 3928 | 8760 | CYP |
| *Streptomyces* | # | Bacteria | 160948224 | 39438 | 2455 | 5097 | CYP |
| *Burkholderia* | # | Bacteria | 242798663 | 44390 | 2893 | 4668 | CYP |
| *Methylobacterium* | # | Bacteria | 57366027 | 16973 | 1461 | 2970 | CYP |
| *Geobacter* | # | Bacteria | 41541244 | 13298 | 1105 | 2025 | CYP |
| *Shewanella* | # | Bacteria | 125027772 | 24049 | 1593 | 1817 | CYP |
| *Coxiella* | # | Bacteria | 11302775 | 4327 | 1583 | 7345 | XCP |
| *Rickettsia* | # | Bacteria | 54282230 | 13149 | 2332 | 6299 | XCP |
| *Streptomyces* | # | Bacteria | 160948224 | 32398 | 2639 | 4838 | XCP |
| *Acinetobacter* | # | Bacteria | 63631253 | 14859 | 2063 | 4531 | XCP |
| *Pseudomonas* | # | Bacteria | 270076122 | 33329 | 2372 | 2052 | XCP |
| *Clostridium* | # | Bacteria | 151714967 | 20453 | 1857 | 1633 | XCP |
| *Deinococcus* | # | Bacteria | 21856631 | 8000 | 662 | 1008 | XCP |
| *Burkholderia* | # | Bacteria | 242798663 | 26149 | 1721 | 973 | XCP |
| *Ruminococcus* | # | Bacteria | 14332320 | 5873 | 527 | 811 | XCP |
| *Bacteroides* | # | Bacteria | 45063795 | 10827 | 724 | 637 | XCP |

# No Rank
