# Supplementary material for: An Efficient Strategy of Screening for Pathogens in Wild-Caught Ticks and Mosquitoes by Reusing Small RNA Deep Sequencing Data
Source: PLoS One. 2014 Mar 11;9(3):e90831. doi: 10.1371/journal.pone.0090831 (PMC3949703; doi:10.1371/journal.pone.0090831)
Supplement: Table S3 — Top 10 genus of Fungi predicted from deep sequencing data of small RNAs. (DOCX) [file pone.0090831.s003.docx]

**Table S3** Top 10 genus of Fungi predicted from deep sequencing data of small RNAs

| **Genus** | **Kingdom** | **Super Kindom** | **Nt-total** | **Match-length** | **Reads number** | **Ratio** | **Sample** |
| --- | --- | --- | --- | --- | --- | --- | --- |
| *Aspergillus* | Fungi | Eukaryota | 190212157 | 46273 | 3012 | 7245 | CYP |
| *Penicillium* | Fungi | Eukaryota | 76043205 | 26120 | 2057 | 6281 | CYP |
| *Pyrenophora* | Fungi | Eukaryota | 34264596 | 13104 | 871 | 1593 | CYP |
| *Thielavia* | Fungi | Eukaryota | 53393454 | 15454 | 1024 | 1458 | CYP |
| *Sporisorium* | Fungi | Eukaryota | 18871829 | 9872 | 620 | 1320 | CYP |
| *Leptosphaeria* | Fungi | Eukaryota | 64328972 | 14444 | 1087 | 1206 | CYP |
| *Nectria* | Fungi | Eukaryota | 25124923 | 10283 | 695 | 1191 | CYP |
| *Candida* | Fungi | Eukaryota | 88678584 | 17972 | 1190 | 1184 | CYP |
| *Zymoseptoria* | Fungi | Eukaryota | 16691196 | 8686 | 537 | 1022 | CYP |
| *Botryotinia* | Fungi | Eukaryota | 61896404 | 14739 | 960 | 1009 | CYP |
| *Leptosphaeria* | Fungi | Eukaryota | 64328972 | 11736 | 1244 | 1283 | XCP |
| *Aspergillus* | Fungi | Eukaryota | 190212157 | 27016 | 1591 | 1180 | XCP |
| *Sarcinomyces* | Fungi | Eukaryota | 41480 | 342 | 56 | 1065 | XCP |
| *Alternaria* | Fungi | Eukaryota | 4359793 | 1929 | 479 | 1034 | XCP |
| *Cladosporium* | Fungi | Eukaryota | 1267372 | 924 | 266 | 761 | XCP |
| *Penicillium* | Fungi | Eukaryota | 76043205 | 13709 | 938 | 686 | XCP |
| *Sporisorium* | Fungi | Eukaryota | 18871829 | 6853 | 512 | 625 | XCP |
| *Pyrenophora* | Fungi | Eukaryota | 34264596 | 8891 | 538 | 412 | XCP |
| *Candida* | Fungi | Eukaryota | 88678584 | 11258 | 818 | 351 | XCP |
| *Cryptococcus* | Fungi | Eukaryota | 1981111 | 1709 | 170 | 322 | XCP |

# No Rank
