# Supplementary material for: An Efficient Strategy of Screening for Pathogens in Wild-Caught Ticks and Mosquitoes by Reusing Small RNA Deep Sequencing Data
Source: PLoS One. 2014 Mar 11;9(3):e90831. doi: 10.1371/journal.pone.0090831 (PMC3949703; doi:10.1371/journal.pone.0090831)
Supplement: Table S4 — Top 10 genus of predicted viruses. (DOCX) [file pone.0090831.s004.docx]

**Table S4 Top 10 genus of predicted viruses**

| **Species** | **Genus** | **Kingdom** | **Super Kindom** | **Nt-total** | **Match-length** | **Reads number** | **Ratio** | **Sample** |
| --- | --- | --- | --- | --- | --- | --- | --- | --- |
| Espirito Santo virus | # | Birnaviridae | Viruses | 6677 | 6361 | 6577 | 2935116137 | *A. sinensis* |
| Nam Dinh virus | # | # | Viruses | 45349 | 12907 | 3367 | 129352109 | *A. sinensis* |
| Cavally virus | # | # | Viruses | 20218 | 2168 | 676 | 2517746 | *A. sinensis* |
| Wolbachia endosymbiont wVitB of Nasonia vitripennis phage WOVitB | # | # | Viruses | 54430 | 2249 | 327 | 167685 | *A. sinensis* |
| Wolbachia endosymbiont wVitA of Nasonia vitripennis phage WOVitA2 | # | # | Viruses | 40003 | 1473 | 256 | 100453 | *A. sinensis* |
| Wolbachia endosymbiont wVitA of Nasonia vitripennis phage WOVitA4 | # | # | Viruses | 21272 | 1305 | 265 | 84029 | *A. sinensis* |
| Culex theileri flavivirus RP-2011 | Flavivirus | Flaviviridae | Viruses | 27786 | 1710 | 110 | 34579 | *A. sinensis* |
| Wolbachia endosymbiont wVitA of Nasonia vitripennis phage WOVitA1 | # | # | Viruses | 66810 | 821 | 142 | 8844 | *A. sinensis* |
| Snake melon asteroid mosaic virus | Sobemovirus | # | Viruses | 345 | 21 | 23 | 5578 | *A. sinensis* |

# No Rank
