# Supplementary material for: An Efficient Strategy of Screening for Pathogens in Wild-Caught Ticks and Mosquitoes by Reusing Small RNA Deep Sequencing Data
Source: PLoS One. 2014 Mar 11;9(3):e90831. doi: 10.1371/journal.pone.0090831 (PMC3949703; doi:10.1371/journal.pone.0090831)
Supplement: Table S5 — GenBank accession numbers of strains used in phylogenetic analysis. (DOCX) [file pone.0090831.s005.docx]

**Table S5 GenBank accession numbers of strains used in phylogenetic analysis**

| **Strain Name** | **GenBank Accession No.** | **Strain Name** | **GenBank Accession No.** |
| --- | --- | --- | --- |
| *Rickettsia sibirica* strain 246 | NR036848 | *Aspergillus niger* strain RPPX1 | KC545869 |
| *Rickettsia sibirica* strain RH05 | HM050271 | *Aspergillus niger* strain RPGX9 | KC545849 |
| *Rickettsia slovaca* str. D-CWPP | CP003375 | *Aspergillus fumigatus* strain s1819 | HQ871898 |
| *Rickettsia slovaca* 13-B | CP002428 | Uncultured *Aspergillus* clone CYP-1 | KC776316 |
| Uncultured bacterium clone HLX-1 | JN866573 | *Aspergillus cristatus* | AB002073 |
| Uncultured Rickettsia clone XCP-1 | KC776315 | *Aspergillus penicillioides* | AB002078 |
| *Rickettsia montana* | U11016 | Uncultured *Aspergillus* clone XCP-1 | KC776317 |
| *Rickettsia aeschlimannii* strain Mc16 | NR026042 | *Aspergillus restrictus* | AB008407 |
| *Rickettsia massiliae* MTU5 | CP000683 | *Aspergillus flavus* strain MJ49 | HM590660 |
| *Rickettsia australis* Phillips | U12459 | *Aspergillus sparsus* | AB002066 |
| *Rickettsia typhi* str. Wilmington | NC006142 | *Ajellomyces dermatitidis* | AF320010 |
| Uncultured bacterium clone DX-68 | JN866592 | Human coronavirus 229E | AGW80930 |
| Uncultured bacterium clone HLX-3 | JN866574 | Murine hepatitis virus strain A59 | NP740616 |
| Uncultured bacterium clone HLC26 | JN866567 | Bat coronavirus strain 61 | AY864196 |
| Uncultured *Coxiella* clone XCP-1 | KC776319 | Avian infectious bronchitis virus | NC001451 |
| *Coxiella* sp. (Rhipicephalus_sanguineus_symbiont) | D84559 | SARS coronavirus | NC004718 |
| *Haemaphysalis longicornis* symbiont 66 | AY342036 | NDV Yunnan | KC776320 |
| Uncultured *Coxiella* clone CYP-1 | KC776318 | Cavally virus isolate C79 | HM746600 |
| *Haemaphysalis longicornis* symbiont A | AB001519 | Nam Dinh virus isolate SZ10618Z | JQ996712 |
| *Coxiella burnetii* RSA 331 | CP000890 | Nam Dinh virus isolate SZ11706Z | JQ996713 |
| *Coxiella burnetii* Dugway 5J108-111 | CP000733 | Nam Dinh virus isolate SZ11826Z | JQ996715 |
| *Coxiella burnetii* CbuG Q212 | CP001019 | Gill-associated virus rdrp | YP001661452 |
| *Legionella* sp. OA32 | AB058916 | Yellow head virus | ACA21302 |
| Equine arteritis virus | NC002532 |  |  |
